# The effect of ecological characteristics on the domestication of sand rice (*Agriophyllum squarrosum*)

**DOI:** 10.7717/peerj.18320

**Published:** 2024-11-27

**Authors:** Cuiyun Chen, Xiaoan Zuo, Xin Zhao

**Affiliations:** 1 Key Laboratory of Ecological Safety and Sustainable Development in Arid Lands, Urat Desert-grassland Research Station, Northwest Institute of Eco-Environment and Resources, Chinese Academy of Sciences, Lanzhou, China; 2 Key Laboratory of Stress Physiology and Ecology in Cold and Arid Region of Gansu Province, Lanzhou, China

**Keywords:** Sand rice, Pioneer species, Seed bank, Seed germination, Domestication

## Abstract

Sand rice (*Agriophyllum squarrosum*) is a pioneer species of annual plant found on mobile dunes in arid and semi-arid areas of China. Its establishment within the community could play a crucial role in the restoration of vegetation in desert environments because the ecological characteristics of sand rice make it well-suited to cope with desertification. Sand rice germinates rapidly when there is sufficient precipitation, and sand burial is beneficial for its germination. After germination, the root system rapidly extends downwards. It has short life cycles, completing the life span in 90 days at drought years. Additionally, sand rice has aerial and soil seed banks, which are suitable for arid ecosystems. Its seeds have high nutrient value of high protein quality and low carbohydrates. These ecological characteristics make sand rice a potentially environmentally friendly crop for addressing future climate change and maintaining food security, especially in desert areas. However it is unknown how ecological advantages affect the *de novo* domestication of sand rice. In this article, we summarize its ecological characteristics and determine optimal growth conditions for domestication and more applications in future.

## Introduction

Desertification is the most important issue in drylands, affecting over 40% of the global land area (Tolba & El-Kholy, 1992; Roy et al., 2024). It leads to decreased productivity and loss of resources and poses significant environmental, economic, and social challenges for residents (Lan et al., 2014). Since 2000, global warming has further exacerbated conditions in deserts, pushing certain arid ecosystems towards becoming hyperarid ecosystems (Huang et al., 2017). Therefore, in the current scenario of climate change and limited resources, it is crucial to understand how to mitigate the degradation of vegetation ecosystems, which is a hot topic for researchers studying desertification (Li et al., 2019; Wang, Liu & Li, 2019). As ecological restoration is a critical component of food security (Abdullah et al., 2022), the driver of vegetation restoration is the community succession of pioneer species (Zhang et al., 2005). Pioneer species utilize soil water better, lower the wind velocity at the soil surface and reduce soil erosion (Li et al., 2007; Jafari et al., 2018).

Sand rice (*Agriophyllum squarrosum*) is the dominant pioneer species at mobile sand dune and is found in the arid and semi-arid areas of China (Chen et al., 2014; Fan et al., 2017). Its distribution area is windy, nutrient-barren, and high solar irradiance (Zhang et al., 2005; Zhao et al., 2016). Sand rice prevents nutrient loss by allometric relationships among the biomasses of different organs (Huang et al., 2009). Its lignified stems can stay in the sand for a long time and are resistant to wind erosion, which protects its small, flat seeds (Zhang et al., 2005; Lan et al., 2014). The seeds are 0.205–1.783 gram m^−2^, which is much higher than that of *Artemisia ordosica* and *Caragana microphylla* (Deng & Liu, 2011). Sand rice seeds are high in nutrients, containing 23.2% protein, 45.0% carbohydrates and 9.7% total fat (Zhao et al., 2021).

High-stress tolerance and nutritional value make sand rice an ideal, environmentally friendly crop of the future (Zhao et al., 2017). Climate change places increasing pressure on agriculture, destabilizing critical cropping systems with biotic or abiotic stresses (Mace et al., 2021). Developing new, tolerant varieties of crops is important for food security. In this article, we summarize the role of sand rice as a pioneer species at mobile dunes, the spatial distribution of seed banks, and its germination character, which will enlighten the current domestication of sand rice.

## Survey methodology

This study comprises an integrative review based on bibliographic searches focusing on sand rice in the desertification of China conducted across the Pubmed, Elsevier and Springer databases. The review includes studies performed on topics such as “Pioneer species’’, “Seed bank”, “Germination”, or “The hindrance of ecological characteristics on the domestication of sand rice”. The main literature found in arid and semi-arid areas are organized according to our focus.

## Review

### Pioneer species

**Figure 1:**
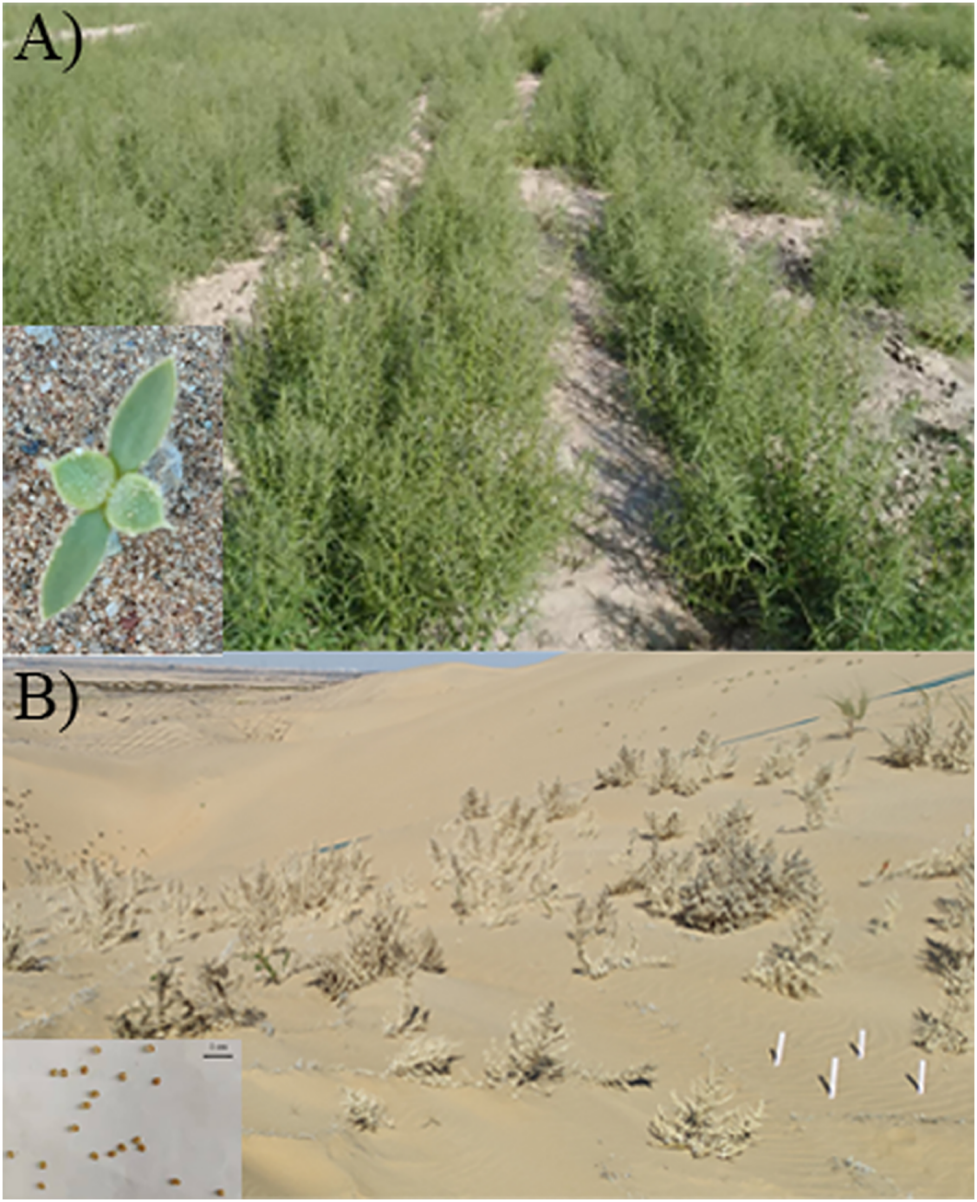
Photos of sand rice in field and wild. (A) Seedlings growing neatly in the field and a newly sprouted seedling. (B) Mature plants grown in the wild and the seeds.

Sand rice has evolved a range of morphological and physiological characteristics to adapt to desert environments. It has tenacious vitality, a short life cycle, high photosynthetic efficiency, and a fast growth and reproduction rate (Li, Chang & Zhao, 1992; Zhang, Zhang & Chang, 2007). Sand rice germinates in May under sufficient precipitation conditions, grows for 2 months at nutritional stage, flowers in August, fruits in September and withers in October (Li, Chang & Zhao, 1992; Qi, An & Ye, 2010) (Fig. 1). But in drought years, the life span only lasts 90 days (Chen et al., 2014). The net photosynthetic rate, water use efficiency increase, while the transpiration rate decreases at a burial depth of 25% of seedling height (Qu et al., 2015). Its seeds germinate quickly when a precipitation event occurs and after germination, the embryonic roots rapidly develop into deep roots to resist wind erosion (Zhang et al., 2005).

Its resistance to cold, drought, barren, wind erosion and sand burial is proved as research continues (Li et al., 2010; Zhao et al., 2013; Qu et al., 2015). The lignified stems and withering plants slow wind speed and reduce sand dune movement (Gao et al., 2015). Sand rice has an increased growth rate, plant height and biomass compared to the control at the burial depth of one-quarter of seedling height (Zhao et al., 2013; Qu et al., 2015). When the burial depth equals the seedling height, its growth rate remains at 94.8%, and plant height and biomass are the same as in the control. Even at the burial depth of 266% of the plant height, the growth rate, plant height and biomass of sand rice are still 14.8%, 58.7%, and 42.9% of the control, respectively (Zhao et al., 2013; Li et al., 2015).

As an indicator of vegetation restoration, the community establishment of sand rice is the starting point (Zhang et al., 2003). After being settled on the mobile sand dune, sand rice grows into dense patches, and the stability of sand matrix is improved (Chang et al., 2003; Huang et al., 2009). Accompanied by the stability of sand matrix, the invasion of other species begins, and the process of community succession and restoration continues (Ma & Liu, 2008; Fan et al., 2017). With the process, species richness and diversity increase, and sand rice changes from pioneer to companion and disappears subsequently (Liu & Wang, 2009; Zhou et al., 2015).

**Figure 2:**
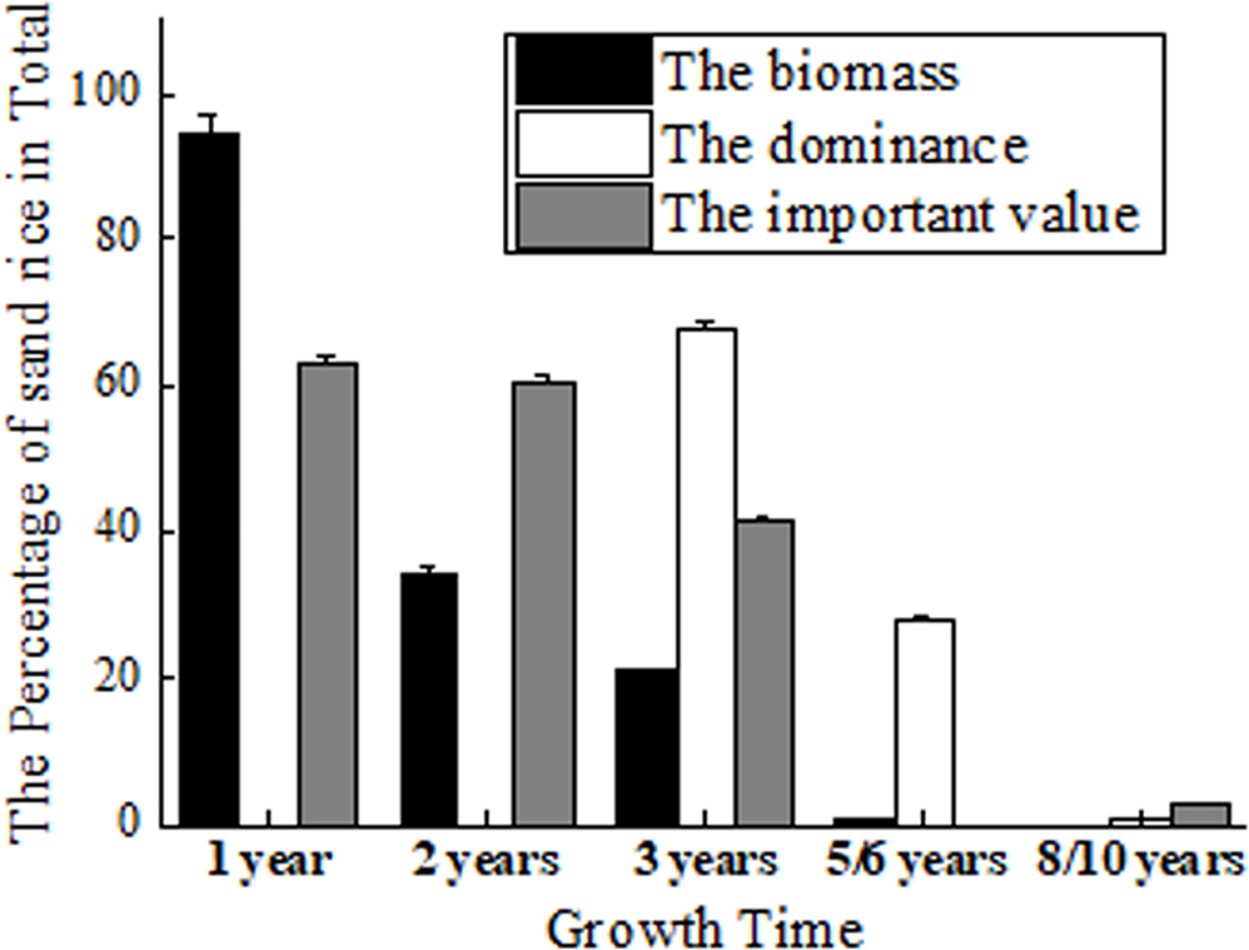
The percentage of sand rice in total by fencing 1, 2, 3, 5/6 and 8/10 years. The data of biomass came from Zhang et al. (2003); the data of dominance came from Zhang et al. (2005); the data of important value came from Li et al. (2007).

Its role as pioneer vegetation has been proven by fencing experiments (Li et al., 2007; Liu & Wang, 2009). The biomass of sand rice accounts for 94.53% of the total biomass in Horqin Sand Land. It maintains 34.11% to the next year and 20.77% to the 3rd year, which declines to 5.08% and 0.66% at the 4th and 5th year (Zhang et al., 2003) (Fig. 2). But Zhang et al. (2005) found that in the 3rd year, sand rice still accounts for 68.08% of the total dominance in Horqin Sand Land. The dominance declines rapidly to 28.02% by year 6 and again to 0.69% by year 10 (Zhang et al., 2005). The difference could be caused by the measurement of the biomass or the total dominance.

At the same time, Li et al. (2007) found that the important values of sand rice are 63.3% and 60.6% in the first and second years of fencing. The important values reduce to 41.5% in the 3rd year and to 2.1% in the 8th year (Li et al., 2007). After fencing 18 years, sand rice has completely disappeared in the sand (Zhang et al., 2005; Liu & Wang, 2009; Ma et al., 2021). In short, sand rice is dominant in the first 1–3 years of fencing. In Hunshandak Sand Land, after 1, 2, 4 and 15 years of fencing, the seed bank frequency of sand rice was 80%, 10.9%, 8.4%, and 0.6%, respectively, indicating that sand rice is dominant in the first year of fencing (Liu & Wang, 2009). Sand rice can be used to green the desert, while simultaneously providing seeds for human consumption. Its rapid growth, ability to germinate at proper burial depth, and resistance to different stresses are important characteristics for the improvement of sand rice.

### Seed bank

Asynchronous seed release increases the survival of the next generation and is an effective strategy to cope with unpredictable environments (Narita & Wada, 1998; Lamont & Enright, 2000; Peters, Martorell & Ezcurra, 2011). Through asynchronous release, sand rice forms two types of seed banks (Liu et al., 2006). One is an aerial seed bank (Gutterman & Ginott, 1994; Gunster, 1994; Narita & Wada, 1998), and the other one is a soil seed bank (Thompson, 1987; Lamont, 1991).

The aerial seed bank includes canopy-stored seeds and released seeds in the air (Liu et al., 2006). Under favorable environmental conditions, the aerial seeds gradually release (Hamilton-Brown et al., 2008), with the majority dispersing from December through March (Liu et al., 2007). Notably, some seeds can remain on the plants for over a year, releasing only during strong winds during the growing season (Ma & Liu, 2008). From September to March, canopy-stored sand rice seeds significantly exceed the released aerial seeds in the Horqin Sand Land (Liu et al., 2006; Ma & Liu, 2008). However, this trend reverses after March, with a marked decline in the total aerial seed bank by May and very few remaining by July in this region (Liu et al., 2006).

In Mu Us Desert, the number of aerial seeds is 4,400 seeds m^−2^ in April, which is significantly more than that of soil seeds. By August, aerial seeds decrease sharply, which is 500 seeds m^−2^ (Gao et al., 2014). Soil seeds have a similar trend to aerial seeds in August.

The soil seed density of sand rice is the highest during first 2 months of seed maturation, then declines from November to March, and relatively stabilizes after March (Ma & Liu, 2008). Most seeds are concentrated at 0–10 cm soil depth from March to June and distribute at 10–50 cm depth after June (Ma & Liu, 2008). Soil seeds are vertically distributed at 0–70 cm depth and increase first and then decrease with increasing burial depth (Bai, Bao & Li, 2004; Liu et al., 2007). The maximum density of sand rice is 843 grains m^−2^ at 10–20 cm depth (Bai, Bao & Li, 2004). However, Liu et al. (2007) found that the highest seed density, 100 grains m^−2^, occurs at a depth of 50–60 cm. This difference might be caused by fresh seeds or persistent seeds (stored for at least 1 year). Deep buried seeds turn into shallow buried or uncovered with dune migration and/or the transition of wind erosion (Liu et al., 2007). Breaking of buried seeds helps long-distance dispersal of sand rice and leads to more extensive seed distribution (Liu & Wang, 2009).

**Figure 3:**
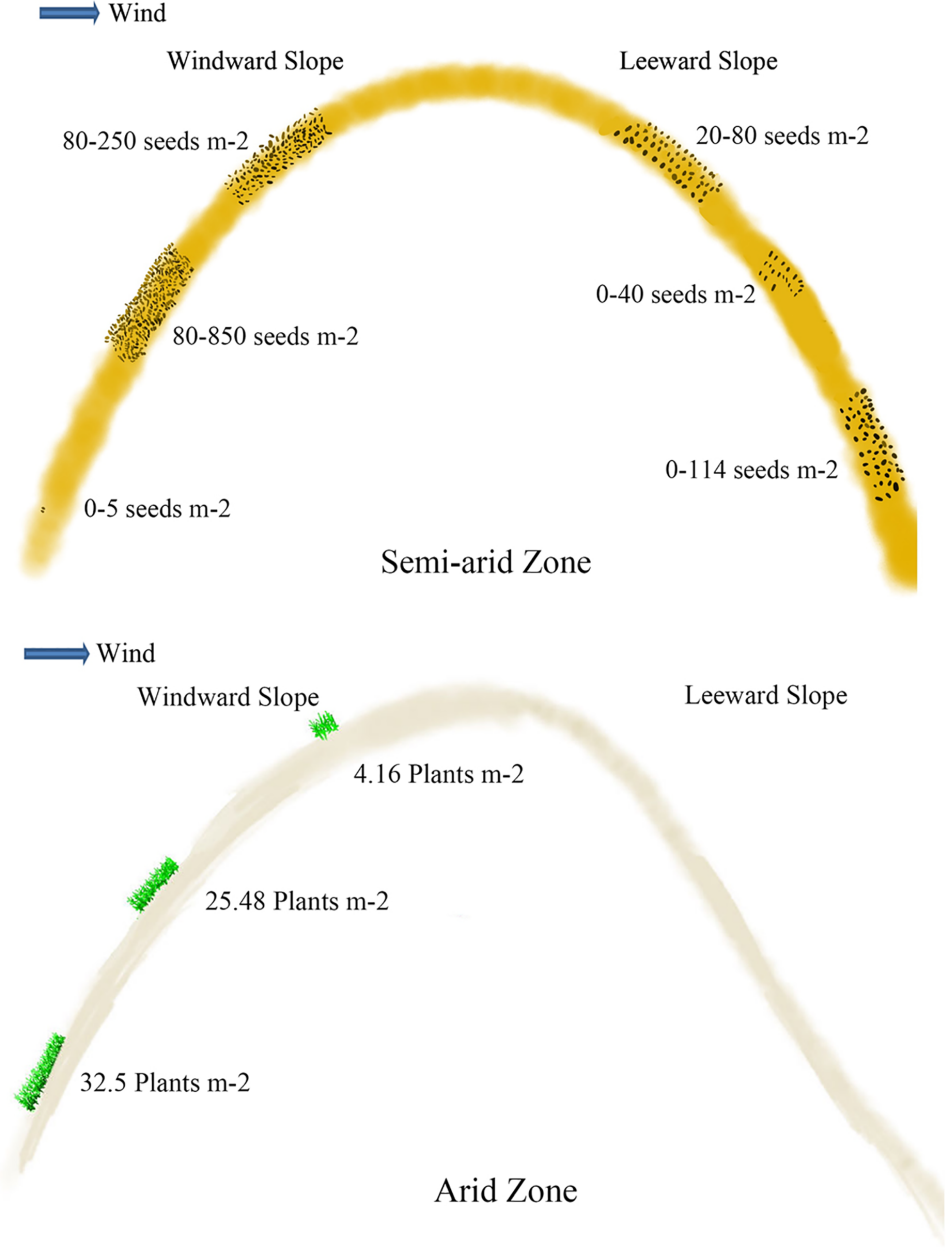
Viable seeds or plants in the persistent seed bank in different positions of the active sand dune in different arid zones. The data of seeds m^−2^ in semi-arid zone came from Bai, Bao & Li (2004) and Liu et al. (2007); the data of plants m^−2^ in arid zone came from Ma et al. (2010).

Soil seeds are affected by different types and positions of sand dunes. Seed density reaches 40.63 grains m^−2^ at 0–5 cm of mobile sand dune, which is 4.3 and 6.5 times higher than that of semi-mobile and semi-fixed sand dunes (Ma et al., 2010; Hu, Ma & Gao, 2010). The seed density at 0–5 cm of fixed sand dune is the lowest. On the windward slope, the density of seeds at 0–5 cm is 152.0 grains m^−2^, which is more abundant than that on the leeward slope (which is 81.6 grains m^−2^) (Bai, Bao & Li, 2004) (Fig. 3). Seed density at 5–10 cm depth on the windward slope is 246.4 grains m^−2^, which is also significantly higher than on the leeward. Seed density at 5–10 cm of mobile sand dunes reaches 12.5 grains m^−2^, which is 4 times higher than that of semi-mobile sand dunes (Ma et al., 2010; Hu, Ma & Gao, 2010). Seeds are undetectable at 5–10 cm layer of fixed and semi-fixed sand dunes (Ma et al., 2010; Hu, Ma & Gao, 2010). At 10–20 cm and 20–30 cm depth of windward slope, seed density is 843.2 and 380.8 grains m^−2^ respectively, which is significantly higher than that of leeward (Fig. 3) (Bai, Bao & Li, 2004).

Soil seeds germinate earlier than aerial seeds (Gao et al., 2014). Different seed germination increases the germination rate of sand rice in extreme environments (Gao et al., 2014). Germination of aerial seeds results in greater plant density than soil seeds because delayed seedling emergence helps to avoid intense competition among seedlings (Gűnster, 1994). However, soil seeds play more important ecological and evolutionary roles in plant populations (Cavieres & Arroyo, 2001; Matus, Papp & Tothmeresz, 2005). Asynchronous seeds (aerial and soil seeds) should be avoided in the domestication of sand rice. The challenge lies in preventing natural seed shedding and promoting synchronized seed maturation.

### Germination

Seed germination is initiated by water imbibition at an appropriate temperature (Ma, Natalia & Abir, 2017). Sand rice germinates intermittently in April and continuously in May. The quick germination pattern in May is beneficial to achieve high population density on the mobile sand dune (Cui et al., 2007). Aerial and soil seed banks have different roles in regulating seed germination (Gao et al., 2014; Fan et al., 2017). Soil seeds germinate earlier in the growing season, while aerial seeds germinate later (Gao et al., 2014). One third of the early germinated seedlings die within 1 month due to different stresses.

Germination of sand rice is affected by environmental factors such as precipitation, temperature, dark/light, and burial depth (Tobe, Zhang & Omasa, 2005; Zheng et al., 2005; Cui et al., 2007) (Table 1). Precipitation is the most important factor in determining seed germination (Tobe, Zhang & Omasa, 2005; Liu, Zhu & Xie, 2022). Precipitation events occur mainly in the summer in the arid zone of Shapotou at the southeast edge of the Tengger Desert of Ningxia, and sand rice germinates in late spring or early summer, when a large proportion of emerging seedlings have sufficient water to survive.

**Table 1:** Germination characteristics of *Agriophyllum squarrosum*.

Sample plot	Seed collection time	Experiment time	Germination rate	Germination conditions	Optimum conditions	Dormancy or not	Authors
Horqin Desert	Oct, 1990	Jun, 1991	17.00%	Sand bury or not before rainfall	0.2 cm sand bury	–	Li, Chang & Zhao (1992)
Horqin Desert	May, 2001	Jun, 2001	15.20%	0–30 cm sand bury	0–5 cm depth	–	Bai, Bao & Li (2004)
Horqin Desert	Autumn 2002	Summer 2003	94.80%	0, 2, 4, 6, 8, 10, 12 cm sand bury depth	2 cm depth	Obligatory dormancy	Li et al. (2004)
Horqin Desert	Autumn 2003	Apr, 2004	42.10% 39.20%	Chilling or dry storage		Stronger innate dormancy	Li et al. (2006)
Horqin Desert	Nov, 2002	May, 2003	91.30%	5/20 °C, 10/30 °C, 20/40 °C;	10/30 °C;	Light or absent	Cui et al. (2009)
				0, −0.1, −0.2, −0.4, −0.8, −1.6 MPa water potentials;	0MPa water potentials;		
				0.5, 1, 2, 4, 8 cm burial depths	0.5 cm		
Horqin Desert	2004–2006	2004–2006	50.30%	Light or dark at 25 °C	Dark	–	Han (2008)
Horqin Desert	1997, 2004–2006	1997, 2004–2006	91.20%	Light or dark at 25 °C	Dark	–	Ma et al. (2010)
Horqin Desert	Autumn, 2003	May, 2005	35%	Chilling or dry storage		–	Ma & Liu (2008)
Horqin Desert	Autumn, 2006	May, 2007	50%	0, 10, 20, 30, 40, 50 mm burial depths	10 mm depth;	No	Luo et al. (2009)
				30, 60, 105 mm irrigation	105 mm		
Horqin Desert	2008	2009	>90%	0.5, 2, 4 cm burial depths;	0.5 cm;	–	Zhao (2009)
			>90%	5, 10, 20 mm water supply	10 mm		
Horqin Desert	Autumn, 2007	2008	85.0%	5, 10, 15, 20, 25 mm precipitation;	25 mm;	Yes	Wang, Yan & Meng (2009)
				0%, 50%, 100% illumination;	0%;		
				0.5, 1, 2, 3 cm burial depths	0.5 cm		
Mu Us Desert	Aug, 2001	May, 2002	33.30%	5/15 °C, 10/20 °C, 15/25 °C, 20/30 °C	20/30 °C in the dark	–	Zheng et al. (2004)
Mu Us Desert	Oct, 2011	Apr, 2012	5.00%	5/15 °C, 10/20 °C, 15/25 °C, 20/30 °C at dark	20/30 °C	Yes	Gao et al. (2014)
Mu Us Desert	Oct, 2012	Jul, 2014	6.67%	0, 9%, 18% soil moistures at 15/25 °C;	0, 9%;	Yes	Gao et al. (2018)
				0, 1, 2, 3, 4 cycles of wetting and drying	4 cycles		
Shapotou	1993, 1994	Jul, 1996	27.40%	Sand bury or not after watering	2 cm depth	No innate dormancy	Wang, Wang & Liu (1998)
Shapotou	2004–2006	2004–2006	23.70%	Light or dark	Dark	–	Han (2008)
Shapotou	1997, 2004–2006	1997, 2004–2006	33.60%	Light or dark at 25 °C	Dark	–	Ma & Liu (2008)
Shapotou	Autumn, 2008	May, 2009	99.20%	0, 1, 2, 3, 4, 6, 8 cm burial depths	1 cm	–	Fan et al. (2010)
Minqin	2004–2006	2004–2006	100%	Light or dark	Dark	–	Han (2008)
Minqin	1997, 2004–2006	1997, 2004–2006	95%	Light or dark at 25 °C	Dark	–	Ma & Liu (2008)
Minqin	Autumn 2010	2011	88%	0, 2, 4, 6, 12, 18, 28, 36 g/ kg NaCl;	18 g/kg threshold;	Yes	Chen, Ma & Wang (2012)
				0, 5%, 10%, 15%, 20%, 30% PEG6000	20% threshold		
Minqin	2010	Apr, 2013	100%	0, 0.0125, 0.025, 0.05, 0.075, 0.1, 0.15 g/mL GA;	0.075, 0.1, 0.15 g/mL GA;	Yes	Wei et al. (2015)
				0, 25%, 50%, 100%, 150%, 200% NaCl;	50% NaCl;		
				0, 10.6%, 16.5%, 21.3%, 25.5% PEG6000	0% PEG		
					6000		
Minqin	Autumn, 2013	2014	15%	0, 0.0125, 0.025, 0.05, 0.075, 0.1, 0.15 g/mL GA;	0.1, 0.15 g/mL GA;	Yes	Tang et al. (2017)
				0, 10.6%, 16.5%, 21.3%, 25.5% PEG6000;	0%PEG6000		
				10, 15, 20, 25, 30, 35 °C;	25 °C;		
				0.5, 1.5, 2.5 cm burial depths	1.5 cm depth		

The effect of temperature on seed germination is achieved by alternating temperature regimes of night and day. The germination percentage of sand rice is 93.78%, 97.82% and 71.8% at 10/20 °C, 20/30 °C and 5/15 °C (night/day) under dark conditions (Zheng et al., 2005). A higher germination percentage (96.7%) at 20/40 °C than at 10/30 °C (91.3%) and 5/20 °C (86.3%) was also found by Cui et al. (2007). However, Gao et al. (2014) found that germination percentage is 47.33%, 75.88% and not detectable at 10/20 °C, 20/30 °C, and 5/15 °C under dark conditions. The lower germination acquired by Gao et al. (2014) is due to fresh seeds compared with the persistent seeds of Zheng et al. (2005) or fresh seeds from another desert of Cui et al. (2007). No matter the difference, temperature regime of 20/30 °C is optimal for sand rice to germinate.

The germination percentage of sand rice is significantly higher in darkness than in light/dark rhythms (Gao et al., 2014). The highest germination percentage is 99.2% at 25/35 °C under dark conditions, while the lowest is 3.57% at 5/15 °C under 215 µmol m^−2^ s^−1^ light conditions (Zheng et al., 2004). In 25 μmol m^−2^ s^−1^ light conditions over a period of 2 to 12 h, germination percentage decreases from 71.30% to 11.97% at 10/20 °C (Zheng et al., 2004). In different deserts, the germination percentages of sand rice are 3.64%, 2.44% and 13.41% under 150 µmol m^−2^ s^−1^ light (Ma et al., 2021). These results indicate that light and/or low temperatures inhibit seed germination.

Seed burial depth is another important factor influencing sand rice germination. Zheng et al. (2005) report 100%, 54.8% and 2.4% germination percentages at burial depths of 0.5, 1, and 4 cm, respectively. Similarly, Cui et al. (2007) observed germination rates of 91.2%, 77.8%, and 21.6% at the same respective burial depths. The highest germination percentage is consistently achieved at a burial depth of 0.5 cm. However, Li et al. (2004) found the highest germination is 95.2% at 2 cm burial depth. This is consistent with the results of Ma et al. (2021). The optimal burial depth might be 0.5–2 cm. This suggests that a relatively shallow burial depth is preferred for efficient seed establishment and emergence.

The germination percentage of fresh sand rice seeds is relatively low, ranging from 12.2–34.1% in semi-arid zones (Deng & Liu, 2011) and 1–10% in arid zones (Liu et al., 2013). However, germination can be significantly improved through appropriate storage conditions. Zeng et al. (2014) demonstrate that storing seeds at 0–5 °C for 25 months increases the germination percentage to 93.3%. Similarly, Gao et al. (2018) found that germination increases from 28% to 100% after 1 month of storage at 30/20 °C (day/night). Dry storage of 1 month at room temperature or prolonged storage at low temperature effectively simulates seed germination of fresh seed of sand rice.

Fresh seeds germinate slowly because of dormancy. Although dormancy has the benefit of reducing the risk that germinated plants die before reproduction due to a lack of water (Wang, Wang & Liu, 1998), it limits the synchronous germination of seeds. Some research implies there is no dormancy in sand rice (Zheng et al., 2004; Tobe, Zhang & Omasa, 2005; Cui et al., 2007). However, environmental deterioration increases the probability of seed dormancy. Seeds sense environmental changes and adjust their dormancy levels to complete germination and seedling establishment (Gao et al., 2018). Sand rice has physiological dormancy (Liu et al., 2013; Ma et al., 2021), even secondary dormancy (Li et al., 2006). Dormancy break is found in seeds stored for 1 month at room temperature (Gao et al., 2018). Additionally, low temperatures, hydration-dehydration cycles, plant hormones, H_2_SO_4_ reagents, and treatment of 1.5 cm burial depth have all been proven effectively to break the dormancy of sand rice (Baskin & Baskin, 2001; Ren et al., 2019). Germination at appropriate temperature, lighting, sand burial and breaking the dormancy are necessary for sand rice improvment.

### The hindrance of ecological characteristics on the domestication of sand rice

Among wild resources, sand rice has been domesticated into food crops because of its high nutritional value (Chen et al., 2014; DeHaan et al., 2016; Zhao et al., 2016). The seeds are consumed by local communities as bean jelly, and were historically used as army provision during the Tang dynasty (Zhang et al., 2018). However, its ecological characteristics, such as gradual seed release, fractional germination, and seed dormancy, have hindered breed improvement. These characteristics must be modified to obtain synchronous and stronger seedlings. Seed germination can reach almost 100% at its optimum growth conditions, including sand burial of 0.5 cm depth at 20/30 °C after fresh seeds are stored at room temperature for 1 month and then kept at 4 °C (Zheng et al., 2004; Tobe, Zhang & Omasa, 2005; Fan et al., 2017). Seed harvesting by machinery during the maturity season remains a challenge due to the issue of gradual seed release.

The yields of sand rice under natural conditions exhibit substantial variability, with reported maximum yields reaching up to 1,281 kg ha^−1^ (Zhang et al., 2018). Induced mutation can improve undesirable traits in sand rice genotypes, thereby accelerating the development of improved cultivars for domestication. High-yielding breeding line of GX-1 and dense planting GX-2 are currently under evaluation (Chen et al., 2014; Zhao et al., 2023). Application of delivery systems bypassing tissue culture may improve agronomic traits of sand rice (Maher et al., 2019; Cody et al., 2023). Concurrently, the implementation of efficient field management practices and the development of innovative harvesting technologies can also contribute to increased sand rice yields (Zhao et al., 2021). Collectively, these strategies ensure that sand rice and its integration as a new, climate-resilient crop can help address food security challenges in China posed by a growing population and a changing climate.

## Conclusions

Based on China’s current social and natural environment, the utilization of sand rice is of great significance. Research on its ecological roles and nutritional value has attracted more and more attention. As a pioneer species, sand rice germinates quickly and in stages. The aerial and soil seed banks are efficient strategies for coping with extreme desert environments. Sometimes, seed dormancy will be induced to avoid the population disappearing under water shortage conditions. However, these characteristics are unsuitable for its domestication. We summarize its optimal growth conditions for the higher yield, including sand burial of 0.5 cm depth at 20/30 °C after fresh seeds stored at room temperature for 1 month, and then kept at 4 °C. As a potential food, sand rice can provide high nutrition to the people of China and other countries. Further funds should be invested in harvesting technology innovation, product promotion, and climate-smart agriculture practices.

## Supplemental Information

10.7717/peerj.18320/supp-1 Supplemental Information 1 list of references of Table 1 in English and Chinese.

## Additional Information and Declarations

### Data Availability

The following information was supplied regarding data availability: This is a literature review.

## References

[ref-100] Abdullah MM, Amjad A, Waleed KZ, Mohtar R, Eidan H, Ali ZA, Anzi BA, Sharma VK, Ma X (2022). Revegetation of native desert plants enhances food security and water sustainability in arid regions: integrated modeling assessment. Science of the Total Environment.

[ref-1] Bai W, Bao X, Li L (2004). Effects of *Agriophyllum squarrosum* seed banks on its colonization in a moving sand dune in Hunshandake Sand Land of China. Journal of Arid Environments.

[ref-2] Baskin CC, Baskin JM (2001). Seeds: ecology, biologeography, and evolution of dormancy and germination.

[ref-3] Cavieres LA, Arroyo TK (2001). Persistent soil seed bank in *Phacelia secunda* (Hydrophyllaceae): experimental detection of variation along an altitudinal gradient in the Andes of central Chile (331S). Journal of Ecology.

[ref-4] Chang X, Yang C, Liu Y, Gao Y (2003). Spatial and temporal dynamics of metapopulation *Agriophyllum squarrosum* in nude sandy habitats. Journal of Inner Mongolia University.

[ref-5] Chen W, Ma R, Wang J (2012). Effect of salt and drought simulated by PEG on seed germination and seedling growth of *Agriophyllum squarrosum*. Agricultural Research in the Arid Areas.

[ref-6] Chen G, Zhao J, Zhao X, Zhao P, Duan R, Nevo E, Ma X (2014). A psammophyte *Agriophyllum squarrosum* (L.) Moq.: a potential food crop. Genetic Resources and Crop Evolution.

[ref-7] Cody J, Maher M, Nasti R, Starker C, Chamness J, Voytas D (2023). Direct delivery and fast-treated Agrobacterium co-culture (Fast-TrACC) plant transformation methods for Nicotiana benthamiana. Nature Protocols.

[ref-8] Cui J, Li Y, Zhao H, Su Y, Drake S (2007). Comparison of seed germination of *Agriophyllum squarrosum* (L.) Moq. and *Artemisia halodendron* Turcz. ex Bess, two dominant species of Horqin Desert. China Arid Land Research and Management.

[ref-9] Cui J, Li Y, Zhao H, Zhang T, Zhao X (2009). Effects of temperature water potential an burial depth on germination of *Agriophyllum squarrosum*. Acta Botanica Boreali-Occidentalia Sinica.

[ref-10] DeHaan LR, Van Tassel DL, Anderson JA, Asselin SR, Barnes R, Baute GJ, Cattani DJ, Culman SW, Dorn KM, Hulke BS, Kantar M, Larson S, David Marks M, Miller AJ, Poland J, Ravetta DA, Rude E, Ryan MR, Wyse D, Zhang X (2016). A pipeline strategy for grain crop domestication. Crop Science.

[ref-11] Deng X, Liu Z (2011). Relative limitation between seed availability and seedling emergence on vegetation restoration of bare patches in degraded grassland. Chinese Journal of Ecology.

[ref-12] Fan S, Baskin CC, Baskin JM, Wang Y (2017). The seed ecology of *Agriophyllum squarrosum*, a pioneer sand dune annual in Central Asia, with particular reference to seed germination. Seed Science Research.

[ref-13] Fan B, Ma Q, Zhang D, An J (2010). Response of seedling emergence of three dominant plant species to soil type and sand burial depth in the southern marginal zone of the Tengger Desert. Arid Zone Research.

[ref-14] Gao R, Yang X, Liu G, Huang Z, Walck Jeffrey L (2015). Effects of rainfall pattern on the growth and fecundity of a dominant dune annual in a semi-arid ecosystem. Plant and Soil.

[ref-15] Gao R, Yang X, Yang F, Wei L, Huang Z, Walck Jeffrey L (2014). Aerial and soil seed banks enable populations of an annual species to cope with an unpredictable dune ecosystem. Annals of Botany.

[ref-16] Gao R, Zhao R, Huang Z, Yang X, Wei X, He Z, Walck Jeffrey L (2018). Soil temperature and moisture regulate seed dormancy cycling of a dune annual in a temperate desert. Environmental and Experimental Botany.

[ref-17] Gunster A (1994). Seed bank dynamics-longevity, viability and predation of seed of serotinous plants in the central Namib Desert. Journal of Arid Environment.

[ref-19] Gűnster A (1994). Variability in life history parameters of four serotinous plants in the Namib Desert. Vegetatio.

[ref-18] Gutterman Y, Ginott S (1994). Long-term protected seed bank in dry inflorescences of *Asteriscus pygmaeus*: achene dispersal mechanism and germination. Journal of Arid Environment.

[ref-20] Hamilton-Brown S, Boon PI, Raulings E, Morris K, Robinson R (2008). Aerial seed storage in *Melaleuca ericifolia* Sm. (Swamp Paperbark): environmental triggers for seed release. Hydrobiologia.

[ref-21] Han X (2008). Physiological and Ecological properties of *Agriophyllum squarrosum*, a pioneer plant for sand control. Master Dissertation of Northwest A&F University, Xianyang, China.

[ref-22] Hu S, Ma J, Gao W (2010). The spatial distribution characteristics of the pioneer plant *Agriophyllum Squarrosum* in mobile dunes. Gansu Science and Technology.

[ref-23] Huang J, Yu H, Dai A, Wei Y, Kang L (2017). Drylands face potential threat under 2 °C global warming target. Nature Climate Change.

[ref-24] Huang Y, Zhao X, Zhang H, Japhet W, Zuo X, Luo Y, Huang G (2009). Allometric effects of *Agriophyllum squarrosum* in response to soil nutrients, water, and population density in the Horqin Sand land of China. Journal of Plant Biology.

[ref-25] Jafari M, Tavili A, Panahi F, Esfahan EZ, Ghorbani M (2018). Wind erosion and regeneration of vegetation cover in arid and semi-arid areas.

[ref-26] Lamont BB (1991). Canopy seed storage and release—what’s in a name. Oikos.

[ref-27] Lamont BB, Enright N (2000). Adaptive advantages of aerial seed banks. Plant Species Biology.

[ref-28] Lan S, Zhang Q, Wu L, Liu Y, Zhang D, Hu C (2014). Artificially accelerating the reversal of desertification: cyanobacterial inoculation facilitates the succession of vegetation communities. Environmental Science & Technology.

[ref-29] Li S, Chang X, Zhao X (1992). *Agriophyllum squarrosum*–study of a pioneer species on mobile sand dunes. Journal of Arid Land Resources and Environment.

[ref-30] Li Y, Cui J, Zhao X, Zhao H (2004). Floristic composition of vegetation and the soil seed bank in different types of dunes of Kerqin steppe. Arid Land Research and Management.

[ref-31] Li H, Gao J, Hu Q, Li Y, Tian J, Liao C, Ma W, Xu Y (2019). Assessing revegetation efectiveness on an extremely degraded grassland, southern Qinghai-Tibetan Plateau, using terrestrial LiDAR and feld data. Agriculture, Ecosystems & Environment.

[ref-32] Li X, Jiang D, Liu Z, Li X (2006). Seed germination characteristics of annual species in temperate semi-arid region. Acta Ecologica Sinica.

[ref-33] Li Y, Ma Q, Zhang D, Ji Y, Zhang J, Liu H (2010). Response of germination rate of three annual plant species to sand burial depths and GA3 in southern marginal zone of the Tengger Desert. Arid Zone Research.

[ref-34] Li Y, Meng Q, Zhao X, Zhang T (2007). Characteristics of species composition and plant diversity in the process of vegetation restoration on moving dunes in the Kerqin Sand land. Actaprataculturae Sinica.

[ref-35] Li J, Qu H, Zhao H, Zhou R, Yun J, Pan C (2015). Growth and physiological responses of *Agriophyllum squarrosum* to sand burial stress. Journal of Arid Land.

[ref-36] Liu Z, Wang X (2009). Functions of canopy-stored seeds in the dune ecosystem: conclusions from *Agriophyllum squarrosum* and *Artemisia wudanica*. Frontier in Biology.

[ref-37] Liu Z, Yan Q, Baskin CC, Ma J (2006). Burial of canopy-stored seeds in the annual psammophyte *Agriophyllum squarrosum* Moq. (Chenopodiaceae) and its ecological significance. Plant and Soil.

[ref-38] Liu Z, Yan Q, Liu B, Ma J, Luo Y (2007). Persistent soil seed bank in *Agriophyllum squarrosum* (Chenopodiaceae) in a deep sand profile: Variation along a transect of an active sand dune. Journal of Arid Environments.

[ref-39] Liu H, Zhang L, Yin L, Zhang D (2013). Effects of temperature, dry storage, and burial on dormancy and germination of seeds of 13 desert plant species from sand dunes in the Gurbantunggut Desert, northwest China. Arid Land Research and Management.

[ref-40] Liu M, Zhu R, Xie H (2022). Responses of germination strategy of *Agriophyllum squarrosum* to rainfall pattern in the Tengger desert. PeerJ.

[ref-41] Luo Y, Zhao X, Huang Y, Zuo X, Wang S, Zhang Y (2009). Seedling emergence of three Chenopodiaceae annuals in response to different sand burial depths and irrigation regimes. Acta Prataculturae Sinica.

[ref-42] Ma J, Liu Z (2008). Spatiotemporal pattern of seed bank in the annual psammophyte *Agriophyllum squarrosum* Moq. (Chenopodiaceae) on the active sand dunes of northeastern Inner Mongolia. China Plant and Soil.

[ref-43] Ma Z, Natalia BV, Abir IU (2017). Cell signaling mechanisms and metabolic regulation of germination and dormancy in barley seeds. The Crop Journal.

[ref-44] Ma Q, Wei L, Chen F, Zhang D, Wang X (2021). Population dynamics of *Agriophyllum squarrosum* along an ecosystem restoration chronosequence in the Tengger desert, China: indication implications for desertification combating. Global Ecology and Conservation.

[ref-45] Ma Q, Zhang D, Jing H, Cheng F, Liu Y, Jin H (2010). Research on distribution characteristics of *Agriophyllum Squarrosum*, an annual pioneer species for desert control.

[ref-46] Mace ES, Cruickshank AW, Tao Y, Hunt CH, Jordan DR (2021). A global resource for exploring and exploiting genetic variation in sorghum crop wild relatives. Crop Science.

[ref-47] Maher M, Nasti R, Vollbrecht M, Starker C, Clark M, Voytas D (2019). Plant gene editing through de novo induction of meristems. Nature Biotechnology.

[ref-48] Matus G, Papp M, Tothmeresz B (2005). Impact of management on vegetation dynamics and seed bank formation of inland dune grassland in Hungary. Flora.

[ref-49] Narita K, Wada N (1998). Ecological significance of the aerial seed pool of a desert lignified annual, *Blepharis sindica* (Acanthaceae). Plant Ecology.

[ref-50] Peters EM, Martorell C, Ezcurra E (2011). The effects of serotiny and rainfallcued dispersal on fitness: bet-hedging in the threatened cactus *Mammillaria pectinifera*. Population Ecology.

[ref-51] Qi K, An X, Ye S (2010). Growth characteristics of *Agriophyllum squarrosum*. Journal of Inner Mongolia Forestry Science and Technology.

[ref-52] Qu H, Zhao H, Zhou R, Li J (2015). Effects of sand burial on survival and photosynthesis characteristics of two Chenopodiaceae annuals. Chinese Journal of Ecology.

[ref-53] Roy P, Pal SC, Chakrabortty R, Chowdhuri I, Saha A, Ruidas D, Islam ARMT, Islam A (2024). Climate change and geo-environmental factors influencing desertification: a critical review. Environmental Science and Pollution Research.

[ref-62] Ren Y, Wei C, He Z, Xu Z, Guo X (2019). Influences of different factors on the vitality and seedling of *Agriophyllum squarrosum*. Journal of Inner Mongolia Forestry Science & Technology.

[ref-54] Tang W, Wei L, Ma Q, Zhang X, Zhang D, Fan B, Chen F, Hu X (2017). Influences of different factors on the germination and seedling of *Agriophyllum Squarrosum*. Journal of Northwest Forestry University.

[ref-55] Thompson K (1987). Seeds and seed banks. New Phytologist.

[ref-56] Tobe K, Zhang L, Omasa K (2005). Seed germination and seedling emergence of three annuals growing on desert sand dunes in China. Annals of Botany.

[ref-57] Tolba MK, El-Kholy OA (1992). The world environment 1972-1992: two decades of challenge.

[ref-58] Wang J, Liu Y, Li Y (2019). Ecological restoration under rural restructuring: a case study of Yan’an in China’s loess plateau. Land Use Policy.

[ref-59] Wang Z, Wang G, Liu X (1998). Germination strategy of the temperate sandy desert annual chenopod *Agriophyllum squarrosum*. Journal of Arid Environments.

[ref-60] Wang L, Yan D, Meng X (2009). Characteristics of seed germination of *Agriophyllum Squarrosum*. Journal of Inner Mongolia Forestry Science & Technology.

[ref-61] Wei L, Ma Y, Ma Q, Zhang D, Ma R, Fan B, Chen F, Hu X (2015). Influence factors analysis of *Agriophyllum squarrosum* germination of mobile sand dunes pioneer plant. Chinese Agricultural Science Bulletin.

[ref-63] Zeng Y, Qi X, Li Y, Wang Y, Wang Y (2014). Optimising the preparation method of seeds of *Agrihyllum squarrosum* prior to staining in tetrazolium solution. Seed Science & Technology.

[ref-64] Zhang C, Zhang Q, Chang J (2007). Diurnal variations of moisture physiology characteristic of a few plants in Hobq Desert. Journal of Nanjing Forestry University.

[ref-65] Zhang J, Zhao H, Cui J, Li Y, Yang J (2003). Biomass of *Agriophyllum squarrosum* community and its function on mobile sand dune in Horqin sand land. Journal of Soil and Water Conservation.

[ref-66] Zhang J, Zhao H, Zhang T, Zhao X, Drake S (2005). Community succession along a chronosequence of vegetation restoration on sand dunes in Horqin Sand land. Journal of Arid Environments.

[ref-67] Zhang J, Zhao P, Zhao J, Chen G (2018). Synteny-based mapping of causal point mutations relevant to sand rice (*Agriophyllum squarrosum*) trichomeless1 mutant by RNA-sequencing. Journal of Plant Physiology.

[ref-75] Zhao M (2009). The physiological and ecological mechanisms of *Agriophyllum Squarrosum* under drought stresses. Doctoral Dissertation of Minzu University of China, Beijing, China.

[ref-68] Zhao P, Li X, Sun H, Zhao X, Wang X, Ran R, Zhao J, Wei Y, Liu X, Chen G (2021). Healthy values and de novo domestication of sand rice (*Agriophyllum squarrosum*), a comparative view against Chenopodium quinoa. Critical Reviews in Food Science and Nutrition.

[ref-69] Zhao H, Qu H, Zhou R, Wang J, Li J, Yun J (2013). Effects of sand burial on growth and physiological process of *Agriophyllum squarrosum* seedlings in Horqin Sand Land of Inner Mongolia, North China. Chinese Journal of Applied Ecology.

[ref-70] Zhao P, Ran R, Sun H, Liu Y, Li X, Wang C, Zhao X, Chen G (2023). Sand rice, a promising future crop for desert and marginal lands in northern China. Grassland Research.

[ref-71] Zhao P, Zhang J, Qian C, Zhou Q, Zhao X, Chen G, Ma X (2017). SNP discovery and genetic variation of candidate genes relevant to heat tolerance and agronomic traits in natural populations of sand rice (*Agriophyllum squarrosum*). Frontiers in Plant Science.

[ref-72] Zhao J, Zhao P, Zhao X, Ma X, Wang Y, Zhou Q, Chen G (2016). Biological character, nutrient value and domestication feasibility of *Agriophyllum squarrosum*. Journal of Desert Research.

[ref-73] Zheng Y, Gao Y, An P, Hideyuki S, Rimmington Glyn M (2004). Germination characteristics of *Agriophyllum Squarrosum*. Canadian Journal of Botany.

[ref-74] Zheng Y, Xie Z, Yu Y, Jiang L, Hideyuki S, Rimmington Glyn M (2005). Effects of burial in sand and water supply regime on seedling emergence of six species. Annals of Botany.

[ref-76] Zhou H, Zhao W, Luo W, Liu B (2015). Species diversity and vegetation distribution in nebkhas of Nitraria tangutorum in the desert steppes of China. Ecological Research.

