# Supplementary material for: The effect of ecological characteristics on the domestication of sand rice (Agriophyllum squarrosum)
Source: PeerJ. 2024 Nov 27;12:e18320. doi: 10.7717/peerj.18320 (PMC11608021; doi:10.7717/peerj.18320)
Supplement: Supplemental Information 1 [file peerj-12-18320-s001.pdf]

Supplement (list of references in English and Chinese)

Bai, W., Bao, X., & Li, L. (2004). Effects of *Agriophyllum squarrosum* seed banks on its colonization in a moving sand dune in Hunshandake Sand Land of China. *Journal of Arid Environments*, 59, 151-157.

Chen, W., Ma, R., & Wang, J. (2012). Effect of salt and drought simulated by PEG on seed germination and seedling growth of *Agriophyllum squarrosum*. *Agricultural Research in the Arid Areas*, 30 (4), 113-119. 陈文, 马瑞君, 王桔红. (2012). 盐和 PEG 模拟干旱胁迫对沙米种子萌发及幼苗生长的影响. *干旱地区农业研究*, 30 (4), 113-119.

Cui, J., Li, Y., Zhao, H., Zhang, T., & Zhao, X. (2009). Effects of temperature water potential an burial depth on germination of *Agriophyllum squarrosum*. *Acta Botanica Boreali-Occidentalia Sinica*, 29(5), 0867-0873. 崔建垣, 李玉霖, 赵哈林, 张铜会, 赵学勇. (2009). 不同环境条件对沙米种子萌发的影响. *西北植物学报*, 29(5), 0867-0873.

Fan, B., Ma, Q., Zhang, D., & An, J. (2010). Response of seedling emergence of three dominant plant species to soil type and sand burial depth in the southern marginal zone of the Tengger Desert. *Arid Zone Research*, 28(6),1038-1044. 樊宝丽, 马全林, 张德魁, 安婧荣. (2010). 腾格里沙漠南缘 3 种优势植物种子出苗对土壤类型和沙埋的响应. *干旱区研究*, 28(6), 1038-1044.

Gao, R., Yang, X., Yang, F., Wei, L., Huang, Z., & Walck Jeffrey, L. (2014). Aerial and soil seed banks enable populations of an annual species to cope with an unpredictable dune ecosystem. *Annals of Botany*, 114, 279-287.

Gao, R., Zhao, R., Huang, Z., Yang, X., Wei, X., He, Z., & Walck Jeffrey, L. (2018). Soil temperature and moisture regulate seed dormancy cycling of a dune annual in a temperate desert. *Environmental and Experimental Botany*, 155, 688-694.

Han X. (2008). Physiological and Ecological properties of *Agriophyllum squarrosum*, a pioneer plant for sand control. Master Dissertation of Northwest A&F University. 韩向东. 固沙先锋植物沙米的生物与生理生态学特征. 2008, 硕士学位论文. 西北农林科技大学.

Li, R., Jiang, D., Liu, Z., Li, X., Li, X., & Yan, Q. (2004). Effects of sand-burying on seed germination and seedling emergence of six psammophytes species. *Chinese Journal of Applied Ecology*, 15(10), 1865-1868. 李荣平, 蒋德明, 刘志民, 李雪华, 李晓兰, 闫巧玲. (2004). 沙埋对六种沙生植物种子萌发和幼苗出土的影响. *应用生态学报*, 15(10), 1865-1868.

Li, S., Chang, X., & Zhao, X. (1992). *Agriophyllum squarrosum*-study of a pioneer species on mobile sand dunes. *Journal of Arid Land Resources and Environment*, 6(4), 63-70. 李胜功, 常学礼, 赵学勇. (1992). 沙蓬--流动沙丘先锋植物的研究. *干旱区资源与环境*, 6(4), 63-70.

Li, X., Jiang, D., Liu, Z., & Li, X. (2006). Seed germination characteristics of annual species in temperate semi-arid region. *Acta Ecologica Sinica*, 26(4), 1194-1199. 李雪华, 蒋德明, 刘志民, 李晓兰. (2006). 温带半干旱地区一年生植物种子的萌发特性. *生态学报*, 26(4), 1194-1199.

Li, Y., Meng, Q., Zhao, X., & Zhang, T. (2007). Characteristics of species composition and plant diversity in the process of vegetation restoration on moving dunes in

the Kerqin Sand land. *Actaprataculturae Sinica*, 16, 54-61. 李玉霖, 孟庆涛, 赵学勇, 张铜会. (2007). 科尔沁沙地流动沙丘植被恢复过程中群落组成及植物多样性演变特征. *草业学报*, 16(6), 54-61.

Luo, Y., Zhao, X., Huang, Y., Zuo, X., Wang, S., & Zhang, Y. (2009). Seedling emergence of three Chenopodiaceae annuals in response to different sand burial depths and irrigation regimes. *Acta Prataculturae Sinica*, 18(2),122-129. 罗亚勇, 赵学勇, 黄迎新, 左小安, 王少昆, 张永锋. (2009). 三种一年生藜科沙生植物出苗对沙埋深度和水分条件的响应. *草业学报*, 18(2), 122-129.

Ma, J., & Liu, Z. (2008).Viability and germination characteristics of canopy-stored seeds of plants in sand dune area. *Chinese Journal of Applied Ecology*, 19(2), 252-256. 马君玲, 刘志民. (2008). 沙丘区植物植冠储藏种子的活力和萌发特征. *应用生态学报*, 19(2), 252-256.

Ma, Q., Zhang, D., Chen, F., Li, Y., & Liu. Y. (2008). Study on seed characters of *Agriophyllum squarrosum*, a pioneer plant on mobile sanddune. Seed, 27(11), 72-76.马全林, 张德魁, 陈芳, 李亚, 刘有军. (2008). 流动沙丘先锋植物沙米的种子特征研究. 种子, 27(11), 72-76.

Tang, W., Wei, L., Ma, Q., Zhang, X., Zhang, D., Fan, B., Chen, F., & Hu, X. (2017). Influences of different factors on the germination and seedling of *Agriophyllum Squarrosum*. *Journal of Northwest Forestry University*, 32(3), 156-161. 唐卫东, 魏林源, 马全林, 张晓娟, 张德魁, 樊宝丽, 陈芳, 胡小柯. (2017). 不同因素对沙蓬种子萌发和出苗的影响. *西北林学院学报*, 32(3), 156-161.

Wang, L., Yan, D., & Meng, X. (2009). Characteristics of seed germination of *Agriophyllum Squarrosum*. *Journal of Inner Mongolia Forestry Science & Technology*, 35(1), 27-29.王丽, 闫德仁, 孟显波. (2009). 沙米种子的萌发特性探讨. *内蒙古林业科技*, 35(1), 27-29.

Wang, Z., Wang, G., & Liu, X. (1998). Germination strategy of the temperate sandy desert annual chenopod *Agriophyllum squarrosum*. *Journal od Arid Environments*, 40, 69–76.

Wei, L., Ma, Y., Ma, Q., Zhang, D., Ma, R., Fan, B., Chen, F., & Hu, X. (2015). Influence factors analysis of *Agriophyllum squarrosum* germination of mobile sand dunes pioneer plant. *Chinese Agricultural Science Bulletin*, 31(7), 18-22. 魏林源, 马彦军, 马全林, 张德奎, 马瑞, 樊宝丽, 陈芳, 胡小柯. (2015). 流动沙丘先锋植物沙米种子萌发影响因素. *中国农学通报*, 31(7), 18-22.

Zhao M. (2009). The physiological and ecological mechanisms of *Agriophyllum Squarrosum* under drought stresses. Doctoral Dissertation of Minzu University of China. 赵敏杰. (2009). *沙米适应干旱环境的生理生态机制*. 硕士论文, 中央民族大学.

Zheng, Y., Gao, Y., An, P., Hideyuki, S., & Rimmington Glyn, M. (2004). Germination characteristics of *Agriophyllum Squarrosum*. *Canadian Journal of Botany*, 82, 1662-1670.
